# Improving patient satisfaction in a multidisciplinary pediatric feeding clinic

**DOI:** 10.1002/jpr3.70067

**Published:** 2025-07-24

**Authors:** Sussette Gonzalez Szachowicz, Linda Cooper‐Brown, Scott Dailey, Emily Garcia, Liyun Zhang, Amy Pan, Rose Lee

**Affiliations:** ^1^ Department of Pediatrics University of Iowa Stead Family Children's Hospital Iowa City Iowa USA; ^2^ Department of Otolaryngology University of Iowa Stead Family Children's Hospital Iowa City Iowa USA; ^3^ Department of Pediatric Gastroenterology Children's Wisconsin Milwaukee Wisconsin USA

**Keywords:** care coordination, family‐centered care, interdisciplinary care, patient feedback, pediatric feeding disorders

## Abstract

**Objectives:**

Pediatric feeding disorders can result from psychosocial dysfunction, poor feeding skills, or medical or nutritional disorders. The primary aim of this study was to evaluate patient satisfaction at the multidisciplinary feeding clinic (MFC) and improve patient satisfaction by reducing patient wait times, improving communication, maximizing clinic space, and expanding inclusivity of our patients and their families in the decision‐making process.

**Methods:**

A survey was created with 14 questions and distributed to all patients who came to the MFC at the University of Iowa. The baseline surveys were administered from September 2021 to November 2021. The following interventions were implemented: reducing time to room a patient, maximizing clinic space, increasing clinical efficiency, and providing more education. Follow‐up surveys were collected from December 2022 to March 2023.

**Results:**

A total of 84 subjects: 41 for the pre‐intervention group and 43 for the post‐intervention group. There were statistically significant differences noted in these categories: the team thoroughly explaining the treatment plan, answering all questions, and getting parents involved in the decision‐making process. Families appreciated the team making the patient feel included throughout the visit. Providing spacious rooms and short wait times were also rated highly.

**Conclusion:**

Overall satisfaction for the MFC at the University of Iowa was rated as highly satisfactory. Areas of further improvement included shortening patient wait times and providing more education for families. This study highlights the continued importance of surveying patients to identify areas of improvement in multidisciplinary clinics.

## Abstract (highlights)

**What is Known:**

- Pediatric feeding disorders are complex and can significantly impact a child's well‐being.
- Team‐based approach to medically complex children has been shown to increase patient satisfaction and enhance quality of care.

**What is New:**

- Multiple factors may influence patient satisfaction, including long wait times during the visit, communication, involvement of patients and families in decision‐making, and understanding of treatment plans.
- Reducing wait times, optimizing clinic space, and providing verbal and written instructions at the end of the visit are the keys to improving overall patient satisfaction.

## 1 INTRODUCTION

Pediatric feeding disorders are defined by the World Health Organization as impaired oral intake that is not age‐appropriate. This may be associated with psychosocial dysfunction, medical, nutritional, or delay of feeding skills. Medical factors that may hinder appropriate feeding include impaired oropharyngeal structure or function, gastrointestinal disorders, neurological, or cardiorespiratory impairments. Children with feeding disorders may develop restrictive eating behaviors, which in turn, can affect growth. Psychosocial factors, including feeding environment and interaction between a child and a caregiver, can also negatively impact feeding progression in the pediatric population.^1^ In 2000, Manikam et al reported that the prevalence of pediatric feeding disorder was approximately 25% with the number closer to 80% in developmentally delayed children.^2^

Given the complexity of pediatric feeding disorders, a multidisciplinary approach including medical, psychosocial, speech pathology, and nutrition is recommended.^1^ There has been an increased need for a multidisciplinary team approach for medically complex children and those with chronic health conditions.^3^ The multidisciplinary clinic approach offers more complete and coordinated care. Institutional costs have been evaluated in different multidisciplinary clinics and shown to be a cost‐effective model of providing patient care.^4^

Improving patient satisfaction is a crucial part of our healthcare that enhances the quality of care.^5^ Communication and the level of information obtained by the physician are positively associated with increased patient satisfaction. And in pediatrics, effective parent–provider communication is associated with parental satisfaction and adherence to treatment.^6^, ^7^, ^8^ Team‐based or multidisciplinary care, described as interprofessional coordination, collaboration, and teamwork, has also been shown to increase patient satisfaction with care.^9^ When patient satisfaction in multidisciplinary clinics, such as the abdominal pain clinic, was compared to a non‐multidisciplinary clinic, the multidisciplinary approach was rated higher for overall patient and family satisfaction.^10^, ^11^

In 2016, Miller and Pentiuk objectively measured patient satisfaction in a multidisciplinary feeding clinic (MFC) using a three‐question questionnaire including subjects such as ease of scheduling an appointment, the team's ability to understand the concerns, and the recommendations from a caretaker's perspective.^9^ To our knowledge, no other studies have been completed to date on patient satisfaction in a pediatric MFC, examining other aspects of multidisciplinary care such as ease of communication, clinic wait times, and involvement in the decision‐making process.

The University of Iowa MFC is comprised of multiple specialists, including a pediatric gastroenterologist, a nurse, a dietitian, a speech pathologist, and a psychologist, who care for children with complex feeding disorders. The primary aim of this study was to evaluate patient satisfaction at the MFC. We also aim to improve patient satisfaction by reducing the wait times from check‐in to meeting the providers, improving communication between the team members, maximizing clinic space, and expanding the inclusivity of our patients and their families in the decision‐making process.

## 2 METHODS

A survey was created with 12 quantitative questions and 2 qualitative questions. Eleven of the quantitative questions were rated on a scale of 1–5, with 1 being *strongly disagree* and 5 being *strongly agree*. The questions asked included domains such as communication after the visit, wait time experience from check‐in to meeting the team, involvement in the decision‐making process, understanding the treatment plan, and all questions being answered by the end of the clinic visit (Appendix SA). The last quantitative question was rated on a scale of 1–10, asking satisfaction with overall care, with 10 being highly satisfied with the care. The two qualitative questions helped elicit open‐ended responses on what could be done to improve the overall clinical experience (Appendix SA). The survey was created based on a valid and reliable survey created by Ygge et al in Sweden.^12^

Surveys were distributed to all patients who came for new or return visits at the University of Iowa's MFC. The surveys were provided in person to the patient and his/her family after the clinic visit. If the patient and/or the patient's family chose to fill out the form, the completed survey was placed by the family in a sealed box. The box remained sealed until the end of the collection cycle. Due to the anonymity of the surveys, there is a possibility that the same family may have completed both the pre‐intervention and post‐intervention surveys.

The baseline surveys were administered from September 2021 to November 2021. Clinic wait times were measured by using the electronic medical records dashboard. The dashboard is utilized by multiple team members, including schedulers, nurses, medical assistants, and physicians. Patient check‐in time (recorded as arrival time) and the time from check‐in to provider entry were recorded.

The following interventions were implemented after the baseline surveys were collected and reviewed: reducing time to room a patient by collecting only the pertinent measurements (vital signs), optimizing available clinic space, increasing clinical efficiency, and providing more education to the families. On arrival at the clinic, if a room was available, patients and their families were taken to the exam room immediately. Medical assistants were trained to obtain only the pertinent metrics, including length, weight, temperature, and oxygen saturation if the patient was on supplemental oxygen. They also verified patients' medications and allergies. This allowed for a more structured approach to each patient encounter, maximizing time available for the clinicians. Clinical space was optimized by providing chairs for everyone in the room, which aimed to minimize crowding when trainees such as medical students and residents were present. The clinical space was further optimized by centering the family in the middle with the clinicians sitting around the family, in our standard clinic room. This encouraged and allowed for face‐to‐face communication with every team member. If the patient was waiting for more than 10 min in a room, the team tried to split the members and start the visit while waiting for the other members to complete the previous visit. At the end of the clinic visit, a team plan was clearly stated in the discharge instructions and printed with the after‐visit summary. For efficiency, one of the team members was in charge of typing out the plan in the after‐visit summary. The plan was verbally reviewed with the parents on the printed after‐visit summary before their discharge from the clinic, all their questions were answered, and a teach‐back method was used to ensure understanding of the plan. Follow‐up surveys were collected in the same manner, from December 2022 to March 2023.

**Figure 1:**
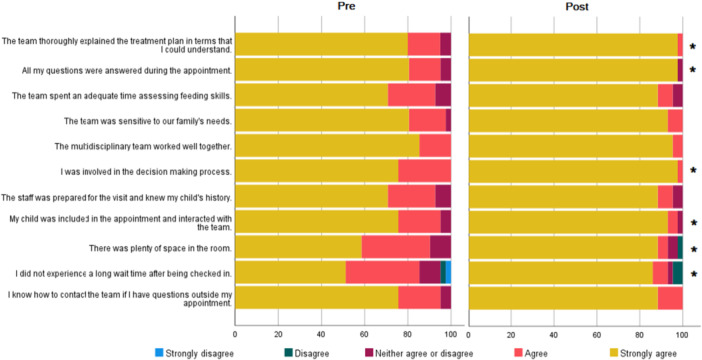
Stacked bar plot presenting the survey results between pre‐ and post‐intervention groups. A Mann–Whitney Wilcoxon test was used to analyze the difference between the pre‐ and post‐intervention groups. There were statistically significant differences noted between pre‐ and post‐intervention groups for the following categories (labeled with * on the figure): team thoroughly explaining treatment plan, all questions were answered, involvement in decision‐making, child was included in the team, there was plenty of space in the room, and not experiencing long wait times.

A Mann–Whitney Wilcoxon test was used to analyze the difference between the pre‐ and post‐intervention groups. A stacked bar plot was used to present the survey results (Figure 1). A two‐sided *p*‐value < 0.05 was considered statistically significant. All data analysis was performed using SAS 9.4.

### 2.1 Ethics statement

The study was designed as a quality improvement project, and as such, it was exempt from institutional review board review.

## 3 RESULTS

There was a total of 84 subjects: 41 for the pre‐intervention group and 43 for the post‐intervention group. Patients were overall satisfied with the medical care provided, with a mean score of 9.51/10 for the pre‐intervention group and 9.76/10 for the post‐intervention group. Figure 1 depicts mean survey results between the pre‐ and post‐intervention groups. There were statistically significant differences noted between the pre‐ and post‐intervention groups for the following categories: the team thoroughly explained the treatment plan, all questions were answered, involvement in decision‐making, the child was included in the team, there was plenty of space in the room, and not experience long wait times. Wait time analysis was divided into two categories: (1) the time between the scheduled appointment and the actual patient check‐in (arrival time), and (2) the time from check‐in to when the provider entered the room. Table 1 depicts median wait times for each category with minimum and maximum wait times for pre‐ and post‐intervention groups. The results were not statistically significant.

**Table 1:** Median wait times for each category with minimum and maximum wait times for pre‐ and post‐intervention groups.

Overall median wait times with minimum and maximum wait times (pre vs. post):	Pre‐intervention (min) (minimum time, maximum time)	Post‐intervention
Time between scheduled appointment time and check‐in time (documented time of patient arrival):	10.50 min (−42.00, 143.00)	6 min (−31.00, 291.00)
Time for the provider to enter the room from check‐in time (documented time of patient arrival):	27.50 min (14.00, 132.00)	25 min (11.00, 211.00)

*Note*: The results were not statistically significant.

Qualitative responses from the pre‐intervention surveys were overall positive, with hand‐selected comments such as “paid a lot of attention to her and helped to feed her,” “providers are attentive.” For areas to improve, comments such as “having a more solid plan,” “larger space for the team to be comfortable.” Post‐intervention qualitative responses included comments such as “everything went well,” “great communication and care,” with no comments on any of the surveys for areas to improve.

## 4 DISCUSSION

Based on previous studies, team‐based approaches to patient care are associated with higher patient satisfaction.^13^ Factors that may influence patient satisfaction include long wait times, such as in an exam room waiting for the provider/team to enter, communication between providers and patients, involvement of patients and families in decision‐making, and understanding of treatment plans. Survey results provided information on different facets of care provided by the multidisciplinary team at the University of Iowa feeding clinic.

Overall satisfaction for the MFC at the University of Iowa was rated as highly satisfactory. However, the identification of areas of improvement, including reducing time to room a patient by collecting only the pertinent measurements, optimizing available clinic space, increasing clinical efficiency, and providing more education to families, allowed for even further improvement in patient satisfaction. The changes that were most effective among pre‐ and post‐intervention groups included explaining the treatment plan thoroughly, answering all questions, involvement in decision‐making, the child was included in the team, and plenty of space in the clinic room. Despite not having statistically significant differences in wait times between the pre‐ and post‐intervention groups, parents perceived an improvement in not experiencing a long wait time. This was likely a result of the intervention to room patients as soon as they arrived at the clinic, if a space was available, as well as having a member of the multidisciplinary team start the evaluation while awaiting the other team members to finish with the previous patient. This may additionally have been a result of increased overall satisfaction in the clinic with the perception that the clinic was sufficiently efficient.

Drawbacks of this study include a small sample size and the fact that the surveys were given after the clinic visit. There might have been more suggestions if the survey had been given at the beginning of the visit. Not all surveys were completed by patients and their families, therefore risking a response bias. Times collected did not include time in the waiting area before being placed in a room and time in the clinic room waiting for the provider, both of which may have impacted results. Given the anonymity of the surveys, we do not have the number of families who may have completed both the pre‐ and post‐surveys. Additional biases to consider include selection bias, whereby the respondents of the surveys may tend to lean toward more positive or negative reviews. Future studies will minimize these biases and address the cost efficiency of the MFC as well as correlate patient satisfaction with patient outcomes.

## 5 CONCLUSION

The study highlights the continued importance of surveying patients and families to identify areas of improvement in multidisciplinary care clinics. Improved physician and patient communication has been shown to improve satisfaction, including understanding of medical diagnoses as well as comprehension of treatment plans and adherence to treatment.^8^ Providing high‐quality patient care through a collaboration between multiple specialists increased patient satisfaction in the MFC. Reducing wait times, optimizing available clinic space, and providing verbal and written instructions at the end of the visit were the keys to improving overall patient satisfaction, with statistically significant differences noted between the pre‐ and post‐intervention groups. This model can be applied to other pediatric multidisciplinary clinics.

## CONFLICT OF INTEREST STATEMENT

The authors declare no conflicts of interest.

## Supporting information

supmat.

SampleSurveyFigureREVISED12.18.24.
