# Supplementary material for: Improving patient satisfaction in a multidisciplinary pediatric feeding clinic
Source: JPGN Rep. 2025 Jul 24;6(4):351–5. doi: 10.1002/jpr3.70067 (PMC12611577; doi:10.1002/jpr3.70067)
Supplement: Supplementary file 1 — supmat. [file JPR3-6-351-s002.docx]

**Supplemental Legends:**

Appendix A - Sample survey with both quantitative and qualitative questions. Quantitative questions rated from 1 to 5 with 1 being strongly disagree and 5 being strongly agree. Overall satisfaction rated on a scale from 1-10 with 10 being the most satisfied.
