# Supplementary figures and images for: Improving patient satisfaction in a multidisciplinary pediatric feeding clinic
Source: JPGN Rep. 2025 Jul 24;6(4):351–5. doi: 10.1002/jpr3.70067 (PMC12611577; doi:10.1002/jpr3.70067)

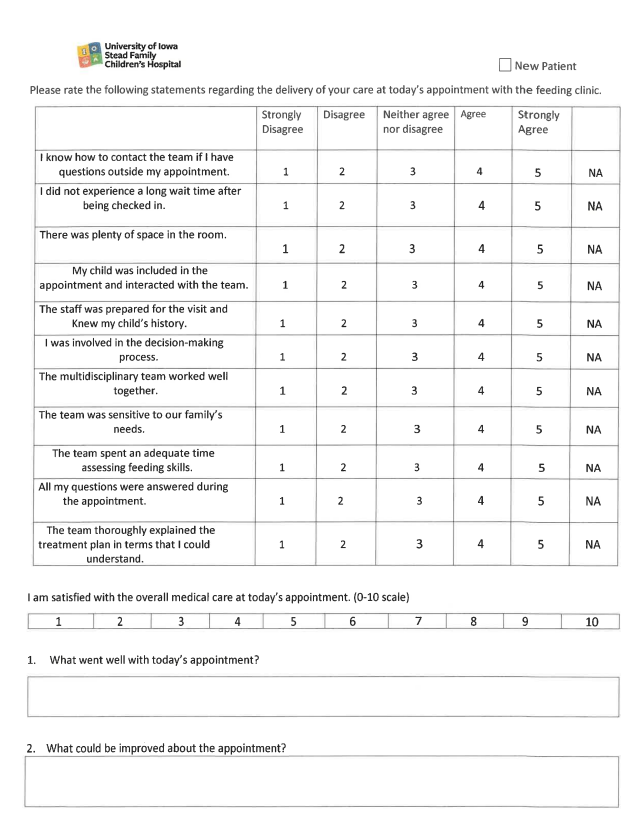

Supplement: Supplementary file 2 — SampleSurveyFigureREVISED12.18.24. [file JPR3-6-351-s001.docx]
